# Looking at China from Abroad: intermediality as a tool for documentary

**DOI:** 10.1007/s40636-023-00261-z

**Published:** 2023-03-16

**Authors:** Cecília Mello

**Affiliations:** Department of Film, Radio and Television, University of São Paulo, São Paulo, Brazil

**Keywords:** Documentary, Animation, Intermediality, Looking china

## Abstract

In 2020, due to the Coronavirus Pandemic, travel to China and around the world was mostly suspended. In the face of such restrictions, the ‘Looking China Youth Film Project’ took a different approach by inviting students from around the world to make documentary short films in remote mode. This meant employing different expressive resources and a mixed-media format, including techniques such as animation, graphics, typography, photographs, and paintings. The aim of this article is to shed light on this pioneering initiative from the point of view of the relationship between intermediality and forms and functions of the documentary genre. It opens with a discussion around the question of intermediality in the audiovisual media, considering how a realist impulse coexists in the cinema with its mixed-media nature, thus bringing together Bazin’s ontology of the photographic image and his case for cinema as an impure form of art. This is followed by a discussion about the recent field of the ‘animated documentary’, both as a practice and as a theoretical debate, which faces the apparent incongruity between film’s unique ability to record reality – which gave rise to the documentary genre, and animation’s creation of a world without any referent in reality. Finally, it considers the experience of the Looking China Youth Film Programme in 2020, with a special emphasis on six documentary films made from a mixture of live images, animation sequences, graphics and screens. The examples analysed allow me to delve into different aspects of the research on cinema and intermediality, including the animated documentary, the influence of the internet in audiovisual media, and the possibilities of a remote practice of film production.

The ‘Looking China Youth Film Project’ is a cultural experience programme sponsored by Beijing Normal University and the Huilin Foundation, and hosted by the Academy for International Communication of Chinese Culture (AICCC). For over ten years, the programme took on students from all over the world, amounting to about 100 every year, who travelled to China for the first time to work from preconceived themes under a general annual theme concerning an aspect of Chinese culture. In 2020, due to the Coronavirus Pandemic, travel to China and around the world was mostly suspended, so the Looking China project took a different approach by inviting students from around the world to make a documentary film of about five to ten minutes in *remote mode*. This meant employing different expressive resources and a mixed-media format, including techniques such as animation, graphics, typography, photographs, and paintings. The general theme for all the films was ‘Farming, Family, Farmer’, and each student filmmaker from around the world would work in pairs or trios and count on the help of a Chinese student producer or producers, located in different parts of the country.

**Fig. 1:**
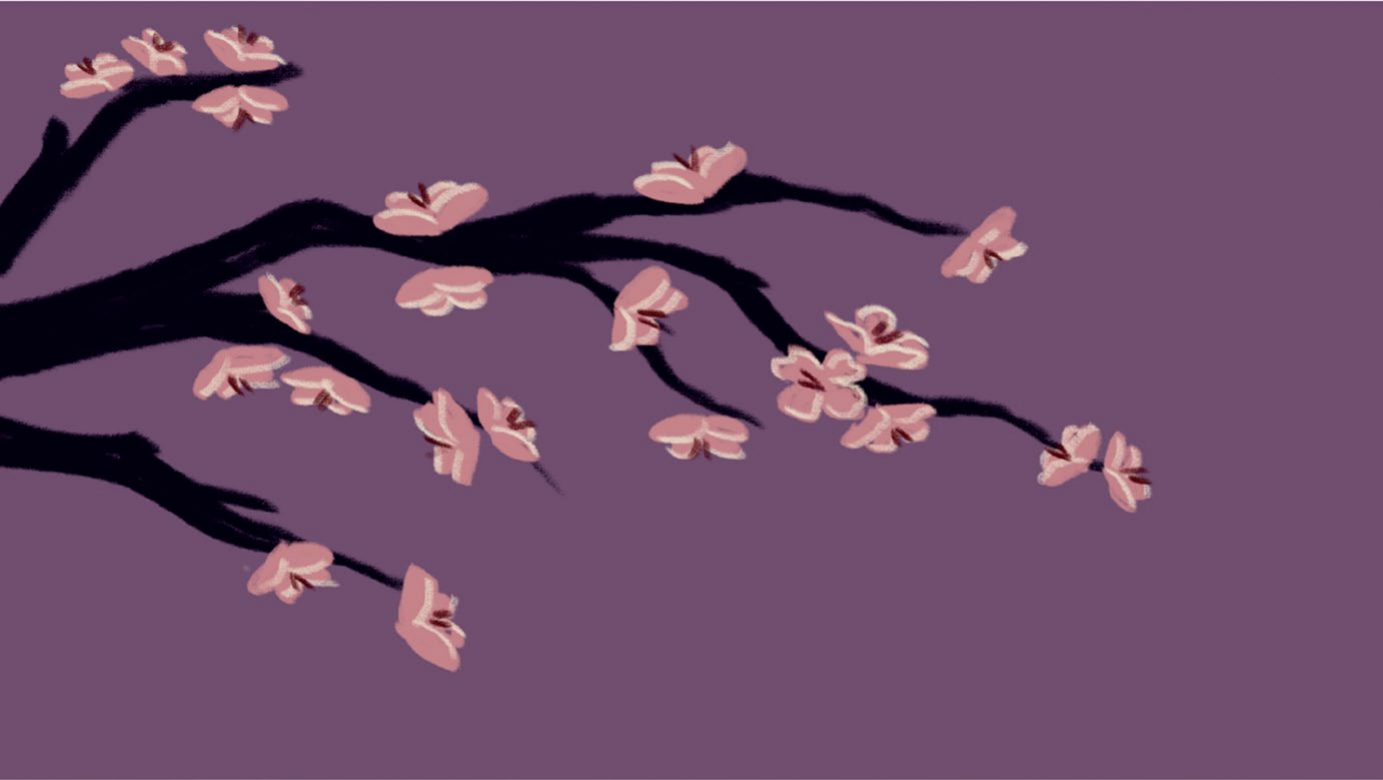
Blessed Peaches (Letícia Midori Sillmann and Lígia Agreste, 2020)

In this article, I will analyse this pioneering initiative from the point of view of the relationship between intermediality and forms and functions of the documentary genre. I am particularly interested in how animation and mixed media can contribute to documentary as the ‘creative treatment of actuality’, as defined by John Grierson (1933, p. 8), revealing other aspects that lie beyond the objective reality and enhancing our understanding of topical issues and stories, landscapes and people. I will first discuss the question of intermediality in the audiovisual media, considering how a realist impulse coexists in the cinema with its mixed-media nature, thus bringing together Bazin’s ontology of the photographic image and his case for cinema as an impure form of art (1967). I will then discuss the recent field of the ‘animated documentary’, both as a practice and as a theoretical debate, which faces the apparent incongruity between film’s unique ability to record reality—which gave rise to the documentary genre, and animation’s creation of a world without any referent in reality. Finally, I will discuss the experience of the Looking China Youth Film Programme in 2020, when I supervised the production of six documentary films made from a mixture of live images, animation sequences, graphics and screens. The examples analysed allow me to delve into different aspects of the research on cinema and intermediality, including the animated documentary, the influence of the internet in audiovisual media, and the possibilities of a remote practice of film production.

**Fig. 2:**
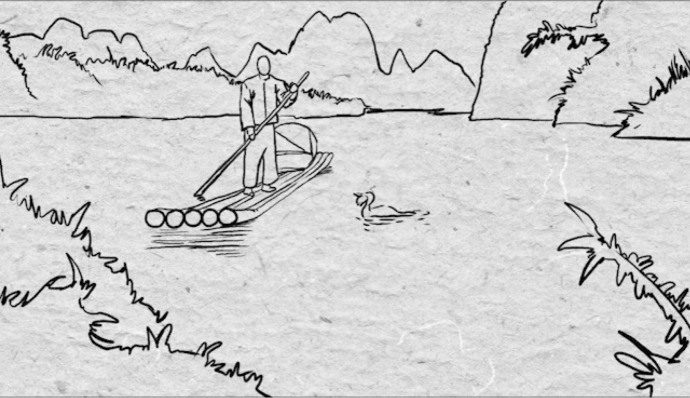
Bird, Man, Water (Sarah Choi, 2020)

## Intermediality: cinema, photography and painting

As I have noted before (see Mello 2019a), the concept of intermediality has become the focus of increased attention in film and audiovisual theory in the past 20 years (Pethő, 2010). It would be wrong, however, to speak of a rehabilitation, since intermediality derives from ‘intermedia’, a word coined by the English poet Samuel Taylor Coleridge in 1812 (1971), but only taken up and defined as a critical term in the mid-1960s by the artist Dick Higgins (1984; 1984). Intermediality, for Higgins, came to describe the activities that took place at the intersection of the arts, which could give rise to new artistic genres such as visual poetry or performance art. Gradually, the term came to designate the interconnections and interferences that occur between different media (Rajewsky 2010). At the same time, its meaning tends to change from the multiple conceptions that involve its root, ‘media’, which makes intermediality a fluid concept, whose heterogeneous nature is now widely accepted. Currently, more contentious than the definition of intermediality is the existence or not of borders, implicit in the use of the prefix ‘inter’, between different media or artistic expressions (Mitchell, 1994).

The importance of the intermedial nature of cinema has been highlighted or minimised since the first writings on the new art in the early twentieth century. It is interesting to note that both positions served a similar purpose of legitimising cinema, either through its approximation with older and more respected artistic traditions or through the search for its specificity as an autonomous art (Pethő, 2010). S.M. Eisenstein (2010) was perhaps the most prolific of voices advocating the dialogue between cinema and other arts in the 1920s and 1930s, bringing it closer to music and architecture. In the 1950s, André Bazin wrote the essay ‘Pour un cinéma impur: défense de l’adaptation’, in which he defended the impure nature of cinema at a time when it was precisely its specificity that most critics and theorists tried to erect and protect in the pages of *Cahiers du Cinéma* (Bazin 1967; De Baecque and Antoine 2001). But even before the ideologues of the *politique des auteurs* and their resistance, for example, to literary adaptations, other authors already showed a concern with the search for the essence or purity of cinema, sometimes seeing its interaction with other arts as a form of a weakening specificity (Arnheim 2006).

Since the 1960s, the debate around cinema’s specificity or impurity has moved on towards a growing number of approaches to intermediality in the field of cinema and audiovisual theory, due both to their absorption by other forms of art and to the multiplication of technological supports with the advent of digital technology, which resulted in a proliferation of new media relationships. The renewed interest in intermediality is also due to the influence of Cultural Studies on film theory and the consequent rejection of the notions of purity and essence, replaced by the concepts of “hybridization”, “transnationalism”, “multiculturalism” and “interdisciplinarity” (Nagib and Jerslev 2013). Cinema, notoriously seen as the meeting point between different arts and sensory regimes, seems to invite cultural hybridization, its intertextuality containing, according to Shohat and Stam (2006), a multicultural nature.

**Fig. 3:**
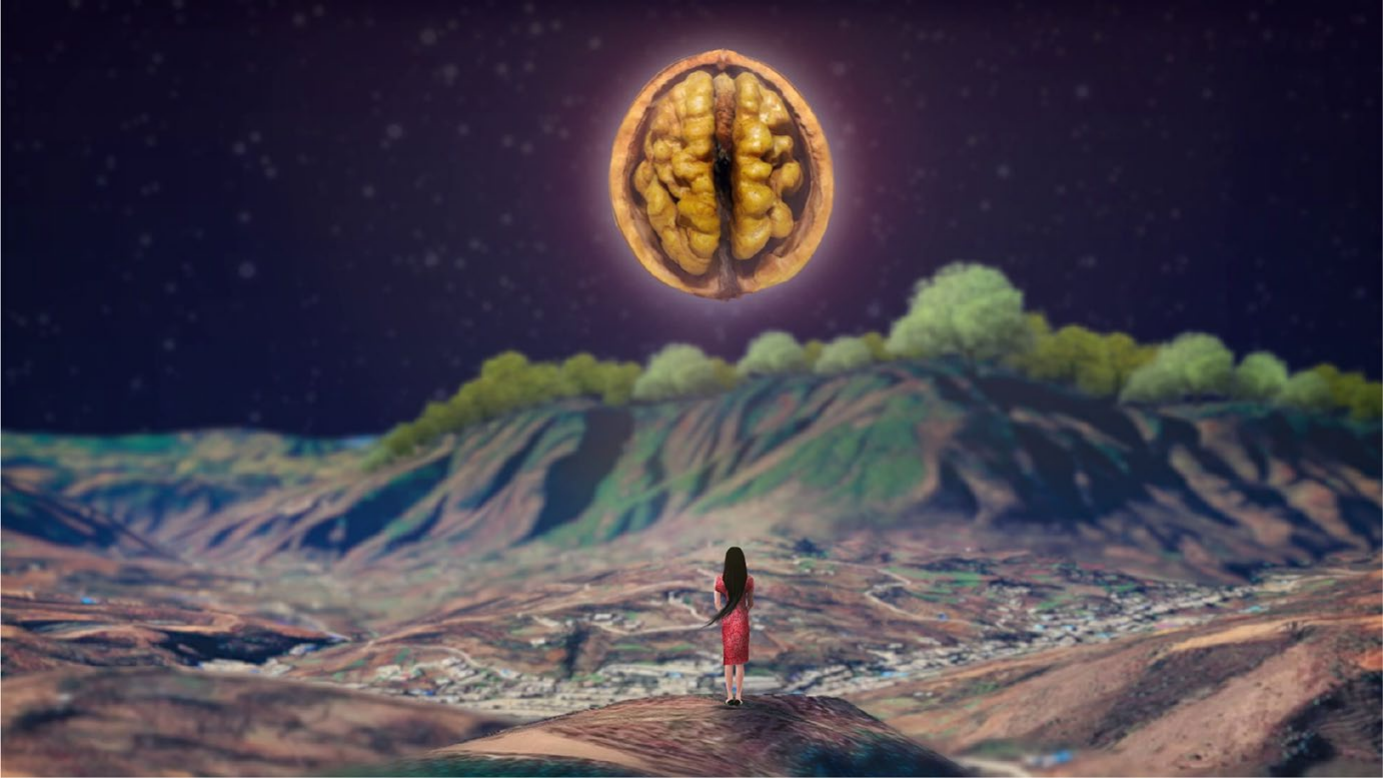
Amber in the Mountains (Atefeh Khademolreza, Sebastien Clermont and Raghed Charabaty, 2020)

Within this growing field, it is possible to detect a number of different angles of approach to cinema’s intermedial nature. These include cinema’s close relationship with music, theatre, dance, performance, and literature. In this article, I am particularly interested in two strands of this debate, the first concerning cinema’s relationship with painting and the second with photography. These, as I will suggest, become interrelated in the practice of animated documentaries, and open up new understandings of film and audiovisual media’s relationship with reality.

**Fig. 4:**
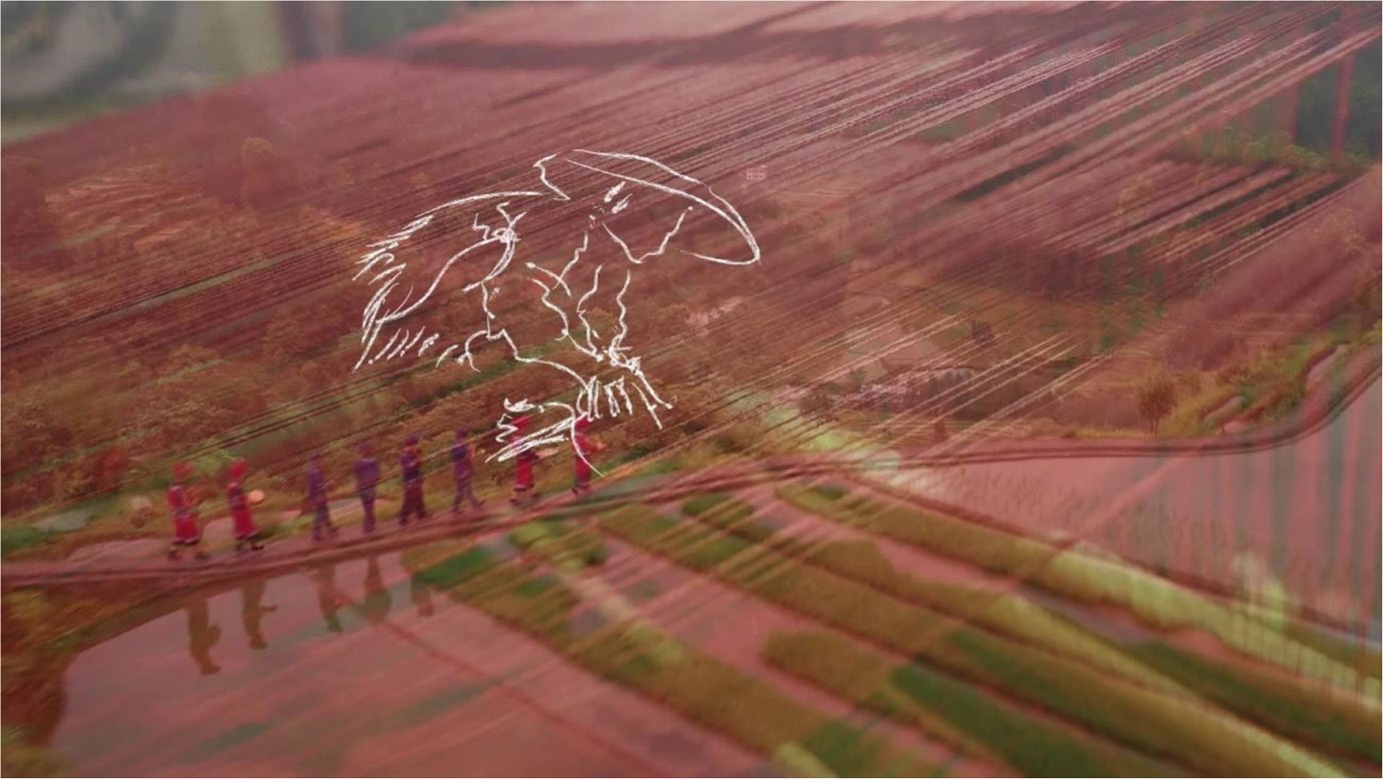
The Broken Branch (Andrea Mock, Nahuel Srnec and Ana Laura Monserrat, 2020)

The relationship between cinema and photography engages head-on with André Bazin’s foundational ‘ontology of the photographic image’, a study that began in 1945 and was published in its final form in 1958. According to Bazin, film, more than any other art, is destined to realism from birth due to its photographic basis, which enables a transfer of ‘the reality from the thing to its reproduction’ (Bazin 1967, p. 14). In *Introduction to Documentary*, Bill Nichols (2017) highlights Bazin’s understanding of cinema as, first and foremost, a document of reality, with the ontology—later translated into semiotic terms by Peter Wollen (1969 1998, p. 86) as ‘indexicality’, seen as offering a visible proof of what the camera recorded. This property is behind the fascination promoted by the cinematographic image and the compulsion generated in the pioneers of cinema towards exploring its ability to display a physical copy of reality, recorded with photomechanical precision on a photographic emulsion, thanks to the passage of light through the lenses. Godard’s famous definition of the cinema as ‘truth 24 frames per second’ is revealing of this compulsion and highlights the photographic origins of cinema, whose movement hides the stillness of the photographic image that guarantees its indexicality (Mulvey 2006).

Conversely, intermedial approaches to the cinematographic art often explore the kinship between cinema and painting. Here, one could evoke another one of Godard’s famous aphorisms, in which the Lumière Brothers are called ‘the last of the impressionist painters’ (see Aumont 1995). In fact, the flat surface of the film can be seen as a canvas, whose illusory three-dimensionality is comparable to the ‘trompe l’oeil’ of painting, with cinema seen as a direct heir of painting (Bonitzer 1985). More recently, intermedial approaches to the cinema have received renewed attention due to the introduction of digital technology in all stages of audiovisual production. The photographic basis of film has been put to the test by the gradual replacement of film by digital technology (Nagib and Mello, 2009), suggesting new interdisciplinary approaches to virtual media and realism and the loss of the ontological or indexical properties of the cinema. An understanding of film as ‘photography 24 times a second’, that is, as an index of reality, has given way to an understanding of film as painting, anchored in the practice of digital compositing (Aumont 1995; Rodowick 2007). This growing technique has been revolutionising the principles of film editing, which until recently had in the ‘shot’ its smallest unit. These days, the increasing ability to remove and insert pixels from a digital or digitised image has turned the pixel into the smallest unit of the visual field of an image and, therefore, of any digital audiovisual work. These codified elements, kept in the computer’s memory, can be manipulated (changed, erased, substituted) as individual units, with help of the appropriate software, thus approximating film to the art and practice of painting. These hybrid images or incrustations caused by digital compositing are materially integrated into the editing, which now incorporates ‘simultaneity’ into ‘succession’ as another one of its foundations. The editor, therefore, builds a combination of images, rather than simply a continuity of shots.

**Fig. 5:**
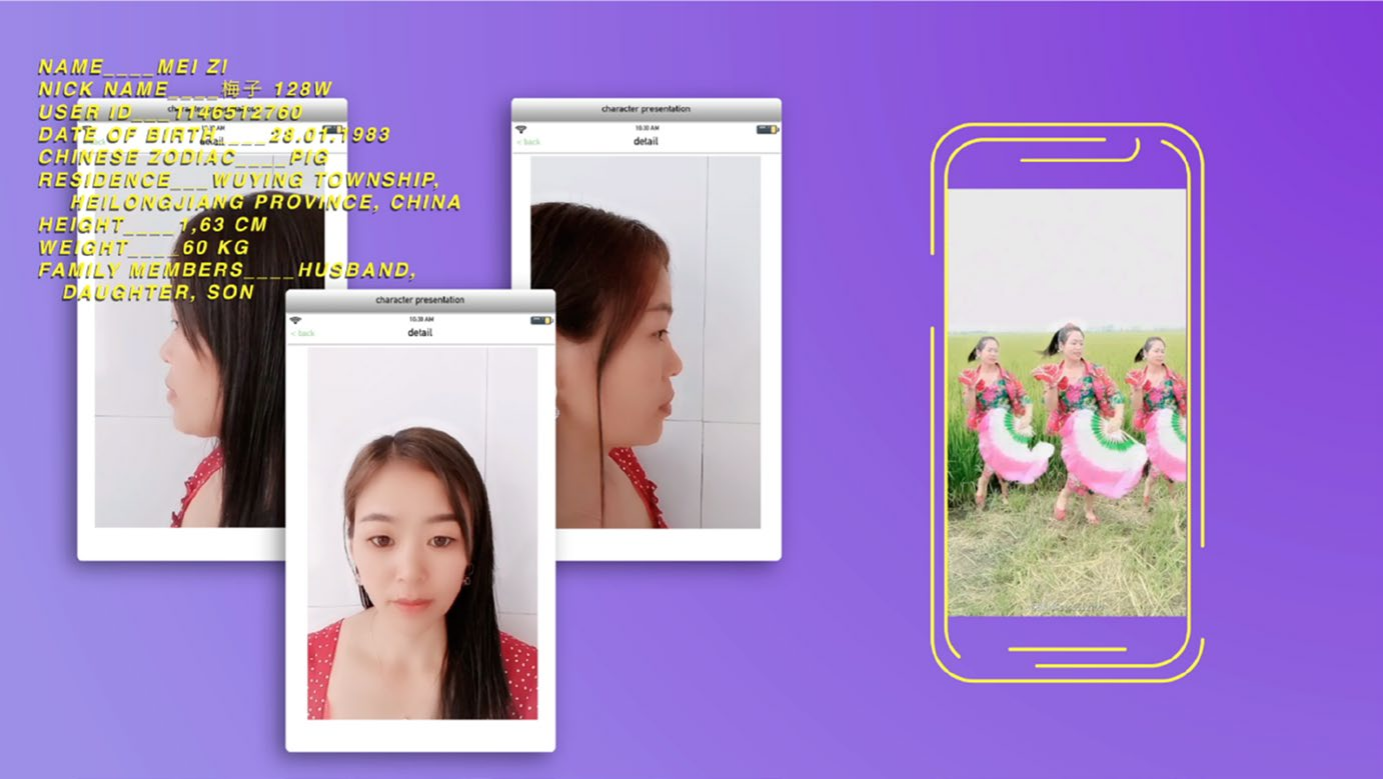
Offbeat Leaders (Celina Duprat, 2020)

## Documentary and animation

Within this debate, Tom Gunning’s essay ‘Moving Away from the Index: Cinema and the Impression of Reality ’(2007) introduces an original contribution by discussing the indexical role of movement in the cinema. According to Gunning, the indexicality of cinema would not be in the image itself, but in the movement it portrays. This validates his assumption of cinema as a branch of animation (following Manovich 2001), and not the other way around. The impression of reality would be the result not of cinema’s pictorial qualities, but rather of its editing, camera movement, actors, objects, and visual rhythms. By the same token, the physical and emotional reactions of a work would be linked to cinema’s ability to both capture and create movement (Gunning 2007, p. 38). Gunning calls this sensation of motion ‘kinesthesia’. Quoting Christian Metz, he goes on to argue that movement is always perceived by the viewer as real, even when portrayed in the flat space of the screen—unlike other visual structures, such as volume, which are often readily perceived as unreal (2007, p. 41). Therefore, the emotional and physical involvement of the spectator with the cinema would be much greater when compared to photography:

We experience motion on the screen in a different way than we look at still images, and this difference explains our participation in the film image, a sense of perceptual richness or immediate involvement in the image. Spectator participation in the moving image depends, Metz claims, on perceiving motion and the perceptual, cognitive, and physiological effects this triggers. The nature of cinematic motion, its continuous progress, its unfolding nature, would seem to demand the participation of a perceiver. (Gunning 2007, p. 42)

Gunning’s powerful case for a new understanding of cinema’s index as movement, and his defence of cinema as a form of animation [he calls the marginalisation of animation resulting from the dominance of a photographic understanding of cinema one of the great scandals of film theory (p. 38)], opens up new theoretical considerations and responds to gradual changes in the world of documentary and animation, traditionally seen as irreconcilable antipodes. Yet, in recent years, the sub-genre or new form known as ‘animated documentary’ (sometimes shortened as anidoc) has shown vigour and reignited the debate around questions of indexicality and intermediality in the cinema.

**Fig. 6:**
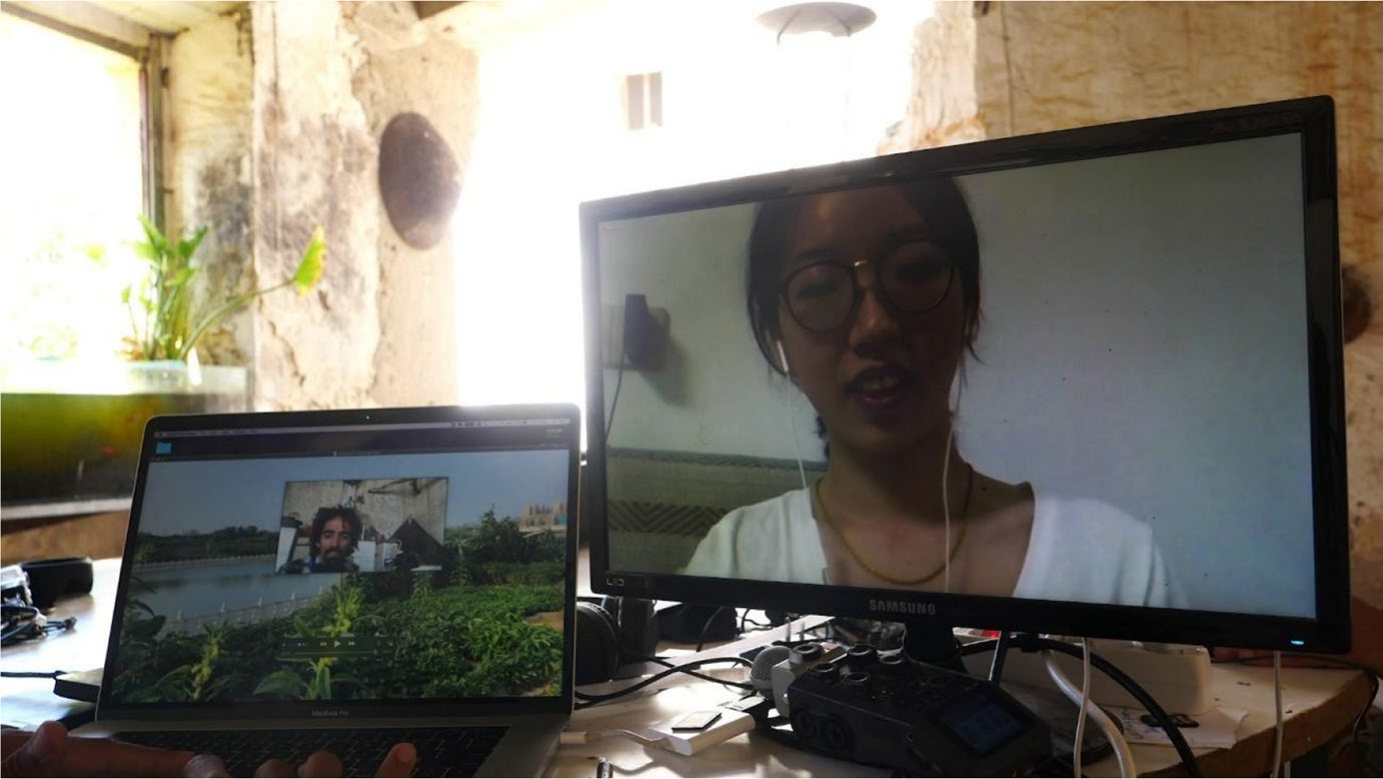
Countries in Countries (Pavel Tavares, 2020)

In her book *Animated Documentary* (2013, Annabelle Honess Roe notes how animation has often been used in non-fictional situations to demonstrate, clarify, and emphasise points, or to portray ideas that live-action films fail to do for one reason or another, such as in Michael Moore’s *Bowling for Columbine* and Frank Capra’s *Why We Fight* series (1942–195).^1^ Roe’s suggestion chimes with Lúcia Nagib’s understanding of intermediality as ‘dissensus’, in Jacques Rancière’s terms, and her suggestion that cinema’s resort to the other arts as expressive means is symptomatic of its own insufficiency. Cinema, Nagib suggests, questions its limits and powers, that is, its autonomy, by embracing impurity, seen as ‘a metaphorical surplus’ that fills the gap of the medium’s own insufficiency (2014, p. 29). In the examples evoked by Roe, live-action or documentary footage could thus be said to welcome animation as a means to overcome their insufficiency.

If those were the first signs of a possible comingling of documentary and animation, it was in the 1990s that the production of what has come to be known as the ‘animated documentary’ began to increase. Even if the majority of animated documentaries are still produced in the short-film format and screened either at festivals or occasionally on television, the increased visibility of the animated documentary has been aided by mainstream cinematic feature film releases such as *Waltz with Bashir* (Ari Folman 2008) and *Chicago 10* (Brett Morgen 2007), as well as the ground-breaking use of digital animation in the BBC’s 1999 prehistoric natural history series *Walking with Dinosaurs*.

Roe notes how the variety of animated documentaries mirrors the variety of animation techniques and subjects for documentaries. She suggests that.

an audiovisual work (produced digitally, filmed, or scratched on celluloid) could be considered an animated documentary if it: (i) has been recorded or created frame-by-frame; (ii) is about the world rather than a world wholly imagined by its creator; and (iii) has been presented as a documentary by its producers and/or received as a documentary by audiences, festivals, or critics. (Roe 2013, p. 3)

In her ground-breaking book, Roe looks at a variety of animated documentary examples and considers the effects of using animation as a representational technique in documentaries, examining the various ways animation is utilised in animated documentaries, including what and how the animation represents, as well as how and why it is chosen to replace live action or the ‘traditional method’. She also examines the ontological distinctions between live action and animation in terms of how they relate to reality. As mentioned before, the visual indexical tie between image and reality, upon which documentary’s claims to truth and evidence so heavily depend, is absent in animation, which falls short of and exceeds indexicality. Yet, Roe suggests that ‘while animation might at first seem to threaten the documentary project by destabilising its claim to represent reality, … the opposite is the case’ (2013, p. 2). She claims that animation alters and broadens the boundaries of what and how we may portray reality by providing new or alternative ways of experiencing the world, in part due to its material distinctions from live-action movies:

[Animation] can present the conventional subject matter of documentary (the ‘world out there’ of observable events) in non-conventional ways. It also has the potential to convey visually the ‘world in here’ of subjective, conscious experience—subject matters traditionally beyond the documentary purview. By releasing documentary from the strictures of a causal connection between filmic and profilmic, animation has the potential to bring things that are temporally, spatially and psychologically distant from the viewer into closer proximity. It can conflate history, transcend geography and give insight into the mental states of other people. (Roe 2013, p. 2)

Finally, Roe remarks that, due to the unconventional interaction between image and reality in animated documentaries, the soundtrack often receives increased attention, raising interesting problems about the way meaning is communicated when standard documentary techniques such as the didactic voice-of-God narration or interview recordings are combined with animated imagery.

## Looking at China from Abroad

These observations into the growing field of animated documentaries introduce some of the central questions raised during the production of the Looking China films in 2020, when travel restrictions were turned into an aesthetic challenge and creative opportunities for young filmmakers around the world. As a participant in the programme representing the University of São Paulo in Brazil, I have been reflecting, over the years, on what characterises the ‘Looking China’ gaze (see Mello 2019b). I have suggested that it could be seen as an at-once travelling, inquisitive and affective gaze. But what happens when the ‘travelling’ gaze can no longer depend on real dislocation as a form of inspiration? And how to solve the problem of making a documentary when you are no longer able to interact in person with your subject and object? How to address and portray a topic extracted from reality without being able to film *in loco*? And even when live-action images were provided to you by the Chinese student producers, how would you edit them as a conventional documentary without imprinting your own gaze into their world?

The challenge was considerable, but in 2020, under the main theme ‘Farming, Family, Farmer’, Looking China’s call for contributions faced up to it:

Farming played a crucial role in ancient China, and it still matters a lot. Due to China’s huge population and an acute shortage of arable land, agriculture has always been a key concern for Chinese people. Apart from its economic implications, farming—and rural life in general—also underscores Chinese people’s appreciation of a peaceful life and harmonious interpersonal relationships. In the post Covid-19 scenario of 2020, where international travel may not be a near possibility, ‘Looking China’ would like to invite young filmmakers from across the world to narrate (from their home bases) mixed-media—animation/graphics/stock footage—stories of China’s farming, farmers and their families.

Under the new circumstances imposed by the pandemic, I was invited to remotely supervise a group of students—including a pair from my department at the University of São Paulo—located in different parts of the world (namely Argentina, Canada, Portugal, Lebanon and Brazil), in addition to their peers located in different provinces in China, from north to south, east to west. Starting in early August 2020, the teams and I employed an array of different resources, from Zoom to Google Meet, from Whatsapp to Wechat, and from QQ to Wetransfer, to communicate and exchange data. We also faced the challenge of working in different time zones, from Vancouver to Buenos Aires, from São Paulo to Toronto in the Americas, and on to Lisbon, Beirut and the official Chinese time, 11 h ahead of Brazil, which made meeting with larger groups tremendously difficult. And even if the live conversation could be circumvented, the exchange of data and rough cuts of films often posed technical challenges. We had rushes, rough cuts and final cuts crossing the distances between us from several different applications, sometimes working well, sometimes less so. And all this with a tight deadline of 18 days for the completion of the film and production of posters, subtitles and other details. This was indeed a great challenge but at the same time a most valuable experience. In the end, the different teams revelled in the exchange of ideas, in the discovery of China’s stories about farming and the relationship between land, nature and people, and in the exploration of audiovisual media’s powers of expression.

The project represented a most valuable experience and achieved beautiful results that deserve scholarly attention. Quite early on, animation was chosen by four out of the six groups selected to work with pre-chosen real topics in different Chinese provinces, ranging from minor animated interventions over live action to full-on animated sequences or the entire film. The two remaining groups, as I will analyse further in the final section of this chapter, chose a different aesthetic alternative to breach the distance separating the filmmakers from their themes: the creative use of multiple screens including desktop images and mobile phone videos.

## Animation and documentary

The documentaries produced in 2020 under my supervision for the Looking China Youth Film Project found different modes to breach the geographical distance and tackle the selected topics, conveying their ideas through multiple instances of intermediality. The first one on this list is *Blessed Peaches*, directed by Letícia Midori Sillmann and Lígia Agreste. In the film, Mr Xu, a Chinese peach farmer, talks about his farm and revisits the mythological origins behind the high-quality peaches of the city of Feicheng in Shandong Province. He narrates an old tale about sacrifice and divine reward while showing his day-to-day routine on his plantation. He had heard the tale from his parents when he was still young, but no written version of the story existed. Rather, it had been told and retold orally through generations.^2^ In this film, the past and the present are articulated through a combination of live-action and animation. Images of Mr Xu recorded on location make up the first and final parts of the film, while the tale is conveyed through a 2D frame-by-frame animation (Fig. 1). The directing duo worked towards a fluid and loose animation style, sometimes veering towards the abstract so that they could easily transition between the moments of mythical storytelling and the contemporary interview. The mixture of animation and live action in this documentary reinforces the importance of oral tales and myths to the trades of the present, revealing the historical, cultural and economic connections between peach farming and the city of Feicheng. It also speaks of how the peach plays an important role in Chinese culture, from the fruit to the peach tree wood, representing good luck and protection. Farming peaches, therefore, is much more than growing fruit for sale and subsistence; it is keeping a tradition alive.

The second documentary on this list is the experimental *Bird, Man, Water*, by Sarah Choi. The challenge faced by Choi and her collaborators was to create a five-minute mixed-media documentary film by using 2-D animation, archival footage, and typography, hoping to shed light on the long-lived tradition of fishing with ospreys in the city of Anzhou, located in the province of Sichuan. The idea, expressed in the film’s proposal, was to ‘mediate the complexities of this ancient tradition’, dating back at least three millennia, but that in our day is ‘on the verge of obsolescence’. Fishing with ospreys involves the long process of taming and training the chicks before they are fit for fishing with humans, a costly and inefficient process, especially in the face of modern mechanized fishing processes. At the same time, even if its eco-friendly status is debatable given the taming of the ospreys and the noose tightened around their necks while fishing, it is a practice that has cultural and historical significance, and that deserves to be treated artistically (Fig. 2).

Choi’s film chooses to employ three different perspectives that make up the three words of the title: an osprey, a fisherman, and the river. The director embraced a three-act structure, depicting three different points of view, but highlighting their symbiotic relationship. Her wish was to present a unique opportunity to consider the intricate interconnectedness between animals, people and Nature. The film employs a limited colour palette made up mainly of grey and beige and uses a minimalistic-style 2D animation from rotoscoping, that is, created by tracing over live-action footage frame by frame. In doing so, Choi promotes passages between the animated medium and reality, a fitting technique that seems to condense, in its at-once faithful and sketchy connection with the live-action footage, the tension between the preciousness and the precariousness of this ancient fishing practice. Finally, the film also employs strokes inspired by the ancient art of Chinese calligraphy, written with ink and a brush. Each act begins with an animation of a brush painting the following Chinese characters: 鸟, 水, 人. The words then transform into animated beings, namely a bird, a river and a fisherman.

The third film to use animation in this group was *Amber in the Mountains*, directed by Atefeh Khademolreza, Sebastien Clermont and Raghed Charabaty. The theme of the film was the traditional cultivation of walnuts in the city of Hezhang in Guizhou Province, located in the south of China. The production of walnuts in the region was greatly stimulated by poverty-alleviation schemes in the past 15 years and has now grown to include sugar-walnut production. The film departs from these facts and imagines the story of a young girl and her father who run a successful sugar walnut business and have planted one of the largest walnut trees in Hezhang. The story is retold as an imagined timeless myth about giving nature love and receiving love back from it. The myth visualises the walnut as a roadmap towards the stars, to one’s dreams and hopes, carved into the shape of the walnut itself (Fig. 3).

The film is split into three sections, namely Past, Present and Future, each animated in a slightly different style, including 2D animation, parallax effect and compositing. The techniques employed are fit to the world of fantasy conjured up by the filmmakers, which includes colourful landscapes, dreamy sequences, a magical, flying salamander and a gigantic walnut that rises in the sky. The film moves chronologically, revealing in the past an arid landscape that will soon become rich and colourful from the power of the rising walnut. In the present, the child and her father grow their walnut tree and turn their love for nature into a unique sugar walnut factory. But the young girl also wishes to escape the dreariness of life in the country, and one day decides to run away to the city. The trope is familiar in the cinema and brings to mind F. W. Murnau’s masterpiece *Sunrise: A Song of Two Humans* (1927). Yet the girl only reaches the city in her dreams, getting lost on the way and being rescued by a flying salamander that falls in love with her sugar walnuts. In the future, the film’s heroine sees the entirety of Hezhang blossoming with sweet walnuts. The sky is filled with stars, and a constellation traces the shape of a shining walnut.

The final film in this section is *The Broken Branch*, by Andrea Mock, Nahuel Srnec and Ana Laura Monserrat. The proposed theme was the Xilankapu technique of Chinese weaving, its connection to the landscape and how it is represented in the fabrics crafted by weaver Zuo Cuiping in Chongqing. The directors intended to make the audience feel the weaving as precious relics inherited and connected to the landscape in Youyang. The film parallels the images of the tension threads on the loom to the great horizon of lines of similar colours in the planting fields and the strips of rivers and lakes that surround the area. By simple animation with white line drawings, small country figures playfully interact with the coloured ‘strings’, while Zuo Cuiping retells her story and explains her weaving techniques, connected to the traditions of the Tujia ethnic group to which she belongs. Animated white traces surround her, defining her outline, her hands and some of the patterns of the Xilankapu (Fig. 4).

## Desktops and mobile phones

The last two films made in 2020 under my remote supervision for the Looking China project also embrace a hybrid format by incorporating, through an intermedial impulse, aesthetic resources from the internet and social media apps for mobile phones. In our day, the internet has created new approaches to audiovisual aesthetics and permeated the culture of moving images in decisive ways. Contemporary desktop films are progressively addressing our everyday experience and our dependence on screens, while the visual and narrative language that has developed from online media platforms has been translated by filmmakers for the big screen, spawning new genres and distorting existing ones. Since the onset of the pandemic, many of our activities have moved to the online environment, increasing the use of screens as tools for communication and creativity. During lockdown periods, the lack of human contact increases, even more, the human need for exchange and sociability, and internet channels such as social media platforms represent a way of sharing our thoughts, our desires and even the minutiae of our daily life.

In *Offbeat Leaders,* by Celina Duprat, the main stars are two farmers and one harvester from Heilongjiang, in the northeast of the country, who find in the application Kwai an outlet for a genuine expression of their hobbies and desires, as well as the promotion of their products. In this context of the mediatization of private life, the main idea of the project was to generate a choral narrative between the three characters, carefully selected to provide a multifocal perspective of what it means to be a farmer in the Northeast of China. These are Liu Xuezhen, a farmer that enjoys singing amidst his fields in hopes of aiding the growth of his crops; Mei, a female farmer who, besides working the land, has practised the traditional Yangko dance of the north of China ever since she was a child; and Wu Di, a charismatic harvester who explores the forest and climbs tall trees to find mushrooms of all sorts.

The film uses both interviews and the diverse material that the characters upload to their personal Kwai accounts. Using this material brings us much closer to them, preventing the distance usually generated by the formal interview. In these videos, they are the ones who decide what and how to shoot and show themselves (Fig. 5). Finally, through the editing, the film employs similar strategies to combine different screens, graphics and written materials into creating a peculiar aesthetic, heavily influenced by mobile phone social apps and the experience of being online on your phone or a desktop. Farming, usually seen as a profession disconnected from the technological advances of the city, appears here to be as connected and dynamic as any other. This shows how Chinese rural culture and people are particularly heterogeneous and diverse.

The last film on the list is *Countries in Countries,* directed by Pavel Tavares, a remote experimental documentary about travelling through the other person’s eyes and body to learn about a different way of farming. The backbone of this metalinguistic documentary is the online encounter between the director Pavel and his Chinese student producer Poli in the year 2020. In the film, Pavel is in Portugal, where he decides to grow his first garden while reminiscing about his experience in Longji, China, where he had been with the Looking China programme in the past. Now, in 2020, he forges a beautiful friendship with Poli, who lives in Zhengzhou, Henan province, and who tells him about her city’s farmers, following their work in the small fields above the city. The contrast between the big city and the farming habits, between urbanisation and the farmland, and between the new apartments and the fields, is explored with great sensibility by the film and through the exchanges between Pavel and Poli. The main themes, therefore, are filtered through the friendship – experienced remotely between two people who share an interest in the knowledge that other, older generations have in connection with the land (Fig. 6).

## Conclusion

Working remotely with the six documentaries analysed in this article highlighted the different possibilities within audiovisual media production in an interconnected world, especially since the advent of digital technologies from the 1990s onwards. Today, it is possible to shoot your film in one or various locations and to edit it remotely, on the opposite side of the globe. It is also possible to create worlds that look real but have no referent in objective reality, or to graft or remove pieces of images from different shots, creating hybrid realities that look real despite their constructed nature. To be sure, these possibilities have always existed, but they have been facilitated tenfold by new advances in technology. One can recall how Lev Kuleshov experimented and theorised, with great wonder, about cinema’s ‘creative geography’, that is, its ability to move between different locations and different times, made to look like a continuous reality through the editing. Today, Kuleshov’s creative geography includes real places and spaces, animated worlds and the worlds within our screens, signalling an unprecedented hybridity that can be seen as both a threat and a path to reality.

In 2020, the ‘Looking China’ initiative represented a fresh ground from which to evaluate both the different gazes and impulses at work in a cross-cultural documentary landscape and how new technologies can allow us to see reality in its many facets. All the six films analysed in this article show how intermediality can enhance the creative treatment of actuality that defines the documentary practice, be it through animation, in all its different guises and forms, or through desktop screengrabs and mobile screens. This brings back the question of intermediality as dissensus, as suggested by Lúcia Nagib (2014), whereby film questions its own lack of autonomy to convey meaning by resorting to other forms of art. Yet it could also be argued that film is, by its own nature, an intermedial phenomenon, whose liberating force allows us to move away from what Henk Oosterling termed the ‘utopia of the Gesamtkunstwerk’, and towards the ‘heterotopia of intermediality’ (2003, p. 38). Oosterling, as well as Pethő (2020), have been crucial voices in advocating for the in-between of intermediality, stressing the need to focus on the epistemological, ethical, and political status of being-in-between, searching for new emancipatory possibilities. From this, it can be concluded that an understanding of cinema as the ‘heterotopia of intermediality’ solves the problem of the film medium’s deficiencies in dramatic expression, which is, in essence, the failure of objective reality itself. Intermediality, therefore, does not mean a betrayal of cinema’s specificity, being quite simply what cinema is. The animated documentary and the desktop film, new tendencies in our current audiovisual landscape, are evidence of new conceptions and new possibilities of the film medium as the heterotopia of intermediality. In the case of the mixed-media Looking China documentaries analysed in this article, intermediality helped us feel connected to China – and the world via China – in wonderful, unexpected ways.

## Data Availability

Not applicable.

## Footnotes

1 *The Sinking of the Lusitania*, a 12-min animation by Winsor McCay, is considered the earliest work in this genre. It depicts the sinking of the RMS Lusitania in 1915 after being hit by two torpedoes fired by a German U-boat, an event for which there is no known film footage. Animation has been utilised in educational and social-advice films since the 1920s, and it is frequently used to demonstrate complex concepts.

2 This is Mr Xu’s tale: Long ago, there was a poor farmer living with his mother. His mother fell ill that year when the weather was so bad that he harvested little from his farming. The doctor could not give him an important medicine because he did not have enough money. He asked if there was any solution. So the doctor told him to cut meat from his own body to substitute that medicine. He did that, cutting his flesh from his thigh. His mother felt better after taking the medicine mixed with her son’s flesh. The seven goddesses witnessed everything, and told the Queen Mother about it. She was deeply moved by the farmer’s movements, so she threw one peach pit which was from her own garden into the yard of that farmer. He found out the peach tree could produce extremely amazing peaches, and this kind of peaches was spreading across the town, making Feicheng a special place for peach planting.
